# Lymphatic Pump Manipulation in Patients with Chronic Obstructive Pulmonary Disease

**DOI:** 10.7759/cureus.4232

**Published:** 2019-03-11

**Authors:** Bruno Bordoni

**Affiliations:** 1 Cardiology, Foundation Don Carlo Gnocchi, Milan, ITA

**Keywords:** copd, lymphatic pump, lymph, osteopathic, inflammation, fascia

## Abstract

Patients with chronic obstructive pulmonary disease (COPD) show a persistent local and systemic inflammatory pattern which stimulates negative remodeling of the airways. Globally, chronic respiratory disease is the third leading cause of death. One of the rehabilitative strategies used to improve the symptoms of COPD patients is the use of lymphatic pump manipulation; this procedure aims to reduce the concentration of pro-inflammatory substances. However, research results relating to this technique are contradictory. This article reviews the mechanisms that determine lymphatic flow, lymphatic lung anatomy, and the lymphatic response to respiratory pathology. Also highlighted is the manual approach to the mediastinum which can be used to improve the lymphatic and inflammatory response in COPD. Finally, new manual strategies have been discussed with which lymphatic flow in patients with COPD can be improved.

## Introduction and background

The pathological factors which lead to chronic obstructive pulmonary disease (COPD) are not fully understood. This disease leads to the gradual restriction of the respiratory airway, with the presence of chronic, local, and systemic inflammation [1]. The respiratory tract is remodeled, from the pulmonary parenchyma to the bronchial tubes; this causes air trapping or hyperinflation, increases in the expiratory reserve volume, and other parameters such as the residual volume and the end expiratory lung volume [2]. There may also be a change in the position and function of the internal and external musculoskeletal structures of the mediastinum. The ribs tend to be in an inspiratory or "inspiratory block" position; this thoracic morphology leads to the so-called "barrel chest" [2]. The diaphragm muscle is flattened and in an inhalation position while the mediastinal fascia, connected to the thorax and shoulders, undergoes an alteration of its intrinsic structure. There is also an increase in the number of myofibroblasts; fascial receptors are transformed into nociceptors and there are several changes in the posture [3]. The cervical tract is in hyperextension, the thoracic vertebral tract assumes a position of hypercifosis, and the shoulders undergo a change in internal rotation [2]. Collectively, these postural and structural changes negatively influence the force expressed by the musculoskeletal system, thus causing a reduced inspiratory pressure [2]. COPD can also cause a range of additional problems, such as cardiac disorders, mood disorders, renal and metabolic diseases, gastroesophageal reflux disease, and intestinal and urogenital disorders [3-5]. According to the Global Burden of Disease (GBD) in 2010, chronic respiratory disease was the third leading cause of mortality in the world; in 1990, it was the fourth leading cause of death in the world [6]. One of the rehabilitative strategies used to improve a patient’s symptoms is lymphatic pump manipulation, used on the thorax or abdomen. This technique aims to reduce the levels of pro-inflammatory substances, thus stimulating lymphatic drainage of the lungs. However, the literature relating to this technique is contradictory [7]. We do know that lymphatic flow is facilitated by the movement of the diaphragm and contraction of the heart, not only at the mediastinal level but also at the systemic level [8-9]. Lymphatic drainage is also regulated by intrinsic stimuli, such as the contraction of lymphatic muscle cells (LMCs) [10-11]. This article reviews the mechanisms that determine lymphatic flow, describes the lymphatic lung anatomy, and reviews the lymphatic response to respiratory pathology. Also highlighted is the manual approach to the mediastinum to improve lymphatic and inflammatory response in COPD. Finally, new manual strategies have been discussed with which lymphatic flow in patients with COPD can be improved.

## Review

The lymphatic system

The main task of the lymphatic system is to remove cellular and interstitial metabolic waste, driving these molecules toward the venous blood or toward the larger lymphatic vessels, so as to keep the cellular systems efficient and to maintain an adequate balance between plasma volume and interstitial volume [12]. The origin of the lymphatic system comes from what is known as "initial lymphatics," small capillaries found in the interstitial fluid. These capillaries are delimited by discontinuous endothelium and basement membranes; this organization creates low resistance to fluids and to transport small molecules, such as viruses and bacteria [12]. The lymphatic capillaries are fixed to the outside by small branches of fibroblastic cells, which form collagen fibrils of type VII. The initial lymphatics widens to create collection ducts consisting of smooth muscle cells and elastic fibers, constructing valves from the endoplasmic cells, which protrude inwards so that molecules coming in cannot return. The lymphatic functional unit is known as a lymphangion. Collecting ducts converge towards the lymphatic nodes (with prenodal collecting lymphatics), which discharge into other collection ducts and nodes (with postnodal collecting lymphatics) [12]. The lymphatic pathways have their own intrinsic active capacity to transport the lymph. Actin filaments have been found in the lymphatic vessels (into smooth muscle cells), which are able to contract and create their own tone. Actin exhibits a transient but constant depolarization due to calcium-dependent chloride currents. This contraction may depend on the sensitivity of actin to feel flow variations (shear stress); the vessels have nerve endings, particularly sympathetic endings, which regulate the contraction of actin filaments [12]. When a lymphangion contracts, the next one fills up; the first will remain empty, while the second will be full. The lymphangions could be compared to a ventricle, as there is a type of pacemaker in each valve, which is sensitive to hydrodynamic variations. When the flow passes to another lymphangion (systole or outflow), the pressure gradient is altered; the latter activates the pacemaker, thus stimulating the contraction of the next functional unit to drain the lymph. Each contraction of a functional lymphatic unit has a measurable time: 1-15 cycles per minute. This could be another intrinsic mechanism responsible for the contractility of the lymphatic vessel [13]. The muscular contraction of the limbs, and of the trunk, is able to passively facilitate lymphatic outflow; other mechanisms which contribute to outflow include: cardiac contractions; arterial pulsations; movements of the diaphragm and postural changes. The speed of the lymphatic flow is approximately 125 mL/h in a person at rest but can increase by a factor of 10 during active movements [12-13]. Another passive mechanism that can manage lymphatic flow is oxygen pressure, particularly at low gradients (25-40 mmHg); if the oxygen pressure is higher, the lymphatic flow slows down. Temperature can also influence the speed of the lymphatic flow; the function of the lymphatic system is optimal at temperatures ranging between 32°C and 37°C, and probably acts via extracellular osmotic changes. Another point to consider is that lymphatic flow will acquire different speeds in different areas of the body [14]. The lymphatic system surrounds and often penetrates all of the organs, bones, and muscles. Most of the lymphatic vessels (under the muscular diaphragm and in a small area above the diaphragm) reach the Chyli cistern. This structure, which exhibits variable morphology, is located under the diaphragmatic area and in front of the first lumbar vertebrae [15]. The lymph collected from the cistern is carried to the thoracic duct, which collects lymph from 80% to 90% of the body. The thoracic duct reaches the jugular vein and the left subclavian vein through the diaphragmatic space of the aortic hiatus, to empty the lymph. In its ascent, it takes the lymph of the musculoskeletal and visceral structures of the mediastinum and turns left at the height of the fifth thoracic vertebra, toward the thoracic outlet. To reach the left veins, it rises above the clavicle by a few centimeters but before reaching the veins, the thoracic duct receives the lymph from the left upper limb and the left cervical area [16-17]. The right lymphatic duct drains the right side of the neck, the right arm, and a small portion of the right chest area. This duct is formed by the confluence of the jugular and subclavian lymphatic trunks, and the right bronchomediastinal trunk; the duct is 10-12 mm long and originates from the right jugulosubclavian junction [17].

Anatomy of the lymphatic system of the lungs

The lymphatic drainage system of the lungs is complex and nonlinear. Lymphatic vessels surround the alveolus but do not penetrate it. The alveoli in healthy lungs are dry; this allows or reduces gaseous exchange through the exhaustive air-blood barrier [18]. When the alveoli are not actively cleaning the lungs, the lymphatic vessels collapse; however, if necessary, in the case of inflammation or infection, the same vessels can significantly increase in size. The lymphatic vessels within the lung can be divided into pleural vessels, interlobular vessels, and intralobular vessels. The latter, with a saccular and tubulosaccular form, include the lymphatic vessels involving the bronchovascular bundles, the perivascular vessels, the peribronchiolar and interalveolar vessels [19]. The interlobular vessels infiltrate deeply into the pulmonary lobule, while the visceral pleural vessels are in continuous contact with the pulmonary parenchyma [19]. The lymphatic vessels inside the lung follow the connective system and are found in greater numbers where connective cells are more concentrated [19]. Drainage from the inside of the lungs and part of the visceral pleura follows back to the bronchial airways via different nodes (intraparenchymal nodes, bronchopulmonary lymph nodes, lower and upper tracheobronchial lymph nodes, and the bronchomediastinal trunk) [20]. All lymphatic drainage from the left lung enters the upper thoracic duct. The right upper lobe is directed to the right paratracheal or parabronchial lymphatic nodes and to the right drainage system. The dorsal area of the right lung reaches the thoracic duct or the inferior ligament of the lung via the inferior tracheobronchial lymphatic nodes. The intermediate lobe can cross and drain into the thoracic duct, or follow the pathways to the right lymphatic area, such as the bronchopulmonary lymph nodes. The lower lobe drains through the lymphatic lymph nodes of the lower ligament of the lung and toward the lymphatic nodes of the paraesophageal and tracheobronchial inferiors [21]. The subpleural lymphatic plexuses are found between the parenchyma and the visceral pleura, and within the visceral pleura; these are always located in the same areas and remain in contact with the lymphatic vessels [18]. Parenchymal lymph drains through the intraparenchymal nodes toward the peribronchial nodes and ends in the left thoracic duct. The visceral pleura may follow different drainage routes, for example, towards the intraparenchymal nodes, or toward the nodes present in the pulmonary hylium (to end up in the left thoracic duct), or by traveling to the pulmonary bases, where the folding of the same hylium forms triangular or pulmonary ligaments [20]. In the lung ligaments, there are many structures of lymphatic vessels and nodes, which serve to carry the visceral pleural lymph toward the lymphatic duct branches [20, 22]. Between the visceral and parietal pleura there is a liquid film, which can be drained from the pleural lymphatic system [22]. The lymph taken from the visceral pleural network is derived from fluids generated by the pulmonary parenchyma and the liquids of the pleural space [23]. The parietal pleura is rich in stomata, circular, or elliptic discontinuities of the mesothelium. These stomata are found in greater densities near the pleural thickening and where the bases of the lung are resting on the diaphragm/endothoracic fascia [22]. The parietal pleural lymph is directed towards the diaphragm muscle, which through its lymphatic drainage system, transports the lymph (together with lymph from the entire abdomen and lower limbs) close to the Chyli cistern and then into the thoracic duct [22]. Lymphatic movement is guaranteed by active, and possibly deep, diaphragm movement, as well as heart contractions; mechanical ventilation (passive stimuli) does not guarantee this lymphatic flow [22, 24]. The lymphatic fate of the lung and the parietal pleura is completely different; lymphatic drainage from the visceral pleura is more similar to that in the lungs. The movement of lymphatic fluid from the parietal pleura depends upon the active movement of the diaphragm and also the heartbeat. The parietal pleura is closely connected with the endothoracic fascia/adipose tissue of the mediastinal cavity, the fascia of which is attached to the epimysium of the deepest intercostal musculature and in a superior manner, to the diaphragm [7]. The endothoracic fascia is rich in lymphatic vessels which receive lymph from the intercostal nodes. Lymph from the last four-seven intercostal spaces is transported into the fascia (via the lymphatic intercostal trunk), then the parietal pleura and finally, towards the cistern of Chyli [17, 25]. The intercostal trunk will carry lymph from the remaining upper spaces toward the base of the thoracic duct, while the portion of the intercostal spaces on the right will empty the lymph into the right lymphatic duct. The lymphatic nodes are surrounded and supported by a network of cells (fibroblasts), which synthesize collagen type III alpha 1 and allow communication with all lymph nodes across the entire body. In this way, each lymphatic node can communicate with several nodes simultaneously [26]. This not only supports the node but prevents its volume from growing excessively in the presence of antigens. Moreover, the network is very resistant. In the case of injury to any lymphatic node, the network would resist, perhaps to maintain a state of immune protection. The same network synthesizes immune cells (cytokines, chemokines) and substances capable of reducing an excessive proliferation of T lymphocytes [nitric oxide (NO)] [26].

How does the lymphatic system behave in the presence of COPD?

The phenomenon of lymphatic angiogenesis occurs when inflammatory or infectious tumour substances are present in the cellular and extracellular environment [19]. In patients with COPD, the number of lymphatic vessels and nodes increases, as does the concentration of the chemokine ligand 21 (CCL21), expressed by endothelial lymphatic cells and one of the A subtypes of the G-protein coupled receptor (GPCR), and its chemokine ligand receptor 7 (CCR7) receptor on activated dendritic cells [19]. This process increases the number of chemokine scavenger receptors 6 (D6) which are synthesized by endothelial cells in order to preserve the CCL21 chemoattractant gradient, facilitate the migration of the CCR7 receptor, and "tag" the immune cells [19]. Lymphangiogenesis is directly correlated with the severity of the disease and is measurable by the value of forced expiratory volume in 1 s (FEV1) [27]. Lymphatic nodes in patients with COPD undergo hypertrophy, particularly at the level of the bronchi up to the bronchioles. In the caudal portion of the trachea, it is possible to find calcified lymphatic nodes or nodes that have been infiltrated by adipose tissue [28]. Dendritic cells (specialized in the capture of antigens) can internalize an antigen by phagocytosis and process the antigen for presentation to T lymphocytes (in lymph nodes), or maintain the antigen on their surface in a native form which is accessible to B lymphocytes (in lymph nodes) specific to that antigen. Their name (dendritic cell) is derived from the particular branched form they assume. Antigen recognition occurs through specific receptors (pattern recognition receptor, PRR; toll-like receptor, TLR) [29]. Conventional dendritic cells (DCs) can stimulate the lymphatic follicle to produce alpha/beta lymphotoxins, which will subsequently induce the dendritic-follicular cell to present the antigen to B-cell lymphocytes. The DCs can indirectly stimulate interleukin-23 (IL-23), thus affecting the production of several cytotoxic lymphatic cells and chemokines, which in turn will stimulate the synthesis of IL-17 (a highly inflammatory cytokine) [30]. The B cells are localized within the lymphatic node, and precisely in the lymphatic follicle (or lymphatic nodule) in the cortical part. In COPD, these B cells are strongly stimulated by B-cell activating factor (BAFF); stimulation results in an increased quantity of circulating B cells, particularly if the patient has a history of smoking. An increase in BAFF indicates an autoimmune disorder and correlates with pathological severity [30-31]. BAFF is known to be stimulated by follicular dendritic cells, monocytes, and macrophages [31]. T lymphocytes stimulate the degradation of elastin, one of the most important causes of emphysema in patients with COPD. This form of degradation is stimulated in turn by the recruitment of IL-17 (synthesized by the indirect stimulation of lymphatic dendritic cells) and interferon-gamma (stimulated by type 1 innate lymphatic cells) [32-33]. Collectively, these events create a vicious circle. Cytokines are soluble molecules and some of the receptors present on surface share the same receptor protein sub-units. The shared soluble gamma chain (sγc) is used as a mechanotransductional tool by interleukins to communicate with multiple receptor types. [34]. Sγc regulates the functional balance of the immune response; this shared chain influences T lymphatic cells, in particular, a subpopulation of these cells known as T regulatory lymphocytes cells (TREG), and inhibits their inflammatory response [35]. In patients with COPD, the levels of detectable sγc are lower than in healthy subjects, indicating that an immunological imbalance/autoimmune is associated with COPD; T cells can no longer be inhibited, thus creating an inflammatory environment that is out of control [35]. Another signal of the constant activation of T lymphocytes and the generally inflammatory status is the reduction in function and quantity of some receptors. T lymphocytes have receptors known as "receptor programmed death" type 1 (PD1) which regulate the half-life of lymphocytes [36]. Studies have shown that the enlargement and neoformation of the lymphatic nodes and vessels reflect the severity of pathology in patients with COPD; there is a close relationship between COPD and pulmonary lymphatic system dysfunction [1, 19, 37-38].

Manual techniques for lymphatic pumping in the mediastinum

One of the rehabilitative strategies employed by physiotherapists and osteopaths is the active manual lymphatic pumping of the mediastinum. The purpose of this technique is to improve pulmonary lymphatic drainage in the presence of respiratory pathology [39]. In this technique, the operator places himself behind the patient; the patient lies on his/her back, placing his/her hands on the anterior area of the mediastinum, from the surface just below the clavicle. The patient then performs a first deep inhalation, while the operator’s hands, during the expiration phase, follow the rib movement in a caudal direction, imposing a push towards the feet. The patient then performs a second inhalation. The operator’s hands hold the position reached by the previous expiratory movement, maintaining a constant pressure on the ribs anteriorly. The patient repeats inspirations, for a total of four inspirations. During this period, the operator’s hands do not move from the chest. At the end of the last inspiratory act, and immediately before the patient expires, the operator quickly removes his hands from the chest [40]. Active manual lymphatic pumping of the mediastinum (AMLPM) was actually conceived by Dr Miller in 1920 and was originally known as the ‘Miller thoracic pump technique.’ However, Dr Miller never treated patients with COPD [41]. Physicians only started using this technique on patients with COPD much later. In the scientific literature, only four articles have investigated or revised the effects of AMLPM, with the method being applied to patients or animals. In 2005, a research team carried out an experiment on five healthy dogs, which were surgically implanted with an ultrasonic flow transducer to measure lymphatic flow in the thoracic duct; results showed that there was an increase in flow in the thoracic lymphatic duct after AMLPM [42]. We cannot task any animal to actively force its breath and are not able to ascertain the strength of the thrusts carried out by the operator. Furthermore, the number of dogs used in this earlier study was too small to be able to draw any firm conclusions and there was no presence of any pathology. The most important and unanswered point relating to this landmark paper, however, is the fact that none of the researchers could identify where the lymph came from, despite the obviously increased rate of flow. In 2008, another research team employed AMLPM on 35 elderly patients with COPD; of these, 18 were enrolled in the experimental group [43]. Only a single session was tested and lasted for just 20 minutes; after this, results were compared with data arising from the spirometric test. Results showed a functional worsening of the airways (greater obstruction). It was not possible to extrapolate the precise causes of this because, in addition to the AMLPM, six other techniques were used for other body areas. In 2009, another study investigated 25 patients with COPD. The operators performed five treatment sessions over a period of five months, with one month intervals; treatments involved AMLPM, passive thoracic pumping on the chest (vibration at each inhalation), and other techniques on different areas of the body [44]. Results demonstrated a worsening of respiratory function in patients. It was likely that patients suffered from an increase in bronchial spasms and an increase in air trapping [44]. Furthermore, some patients complained of post-treatment chest pain. Most recently, in 2015, a systematic review highlighted the lack of positive effects of manual treatment upon respiratory function in the mediastinum (FEV1, forced vital capacity, residual volume) or upon the lymphatic system [8].

AMLPM and COPD: can we do better?

Patients with COPD suffer from a range of different comorbidities, including osteoporosis, arthritis, and chronic pain. The cost-sternal and cost-vertebral joints are more rigid and calcified in patients with COPD than people without the disease [4, 45]. Due to their respiratory condition, these patients have difficulty remaining on their backs for 20-30 minutes at a time and are often associated with heart failure and dyspnea [46]. As described earlier in this article, the rib cage is rigid, in an inspiratory attitude and shows postural changes [2]. Attempts to stimulate the active and passive contribution of the structure of the lymphatic vessels and nodes, using a manual approach, in a situation where the same structures are altered in terms of form and function, are therefore extremely unrealistic. The thoracic duct lies deep in the mediastinum, as previously described [16]. At present, there is no evidence to support the fact that the application of AMLPM is able to directly stimulate the thoracic duct. The manual pumping approach to stimulate lymphatic drainage from the lungs does not follow the information relating to the diversified drainage between the lungs and the parenchyma, the visceral and parietal pleura. The breathing of patients with COPD, and in particular those with medium and severe forms of the disease, cannot perform a deep inhalation due to diaphragmatic dysfunction and reduced parenchymal elasticity; consequently, trying to stimulate lymphatic outflow with deep breaths is simply not feasible. Lymphatic pumping would stimulate the elasticity of the smooth muscles in the bronchi, which are predisposed to prevent a widening of the bronchi during inflation (and not to restrict the bronchi during expiration), so as to maintain morphology throughout inhalation [47]. These imposed vibrations could cause a further narrowing of the bronchi, as some studies have highlighted [43-44]. We know that it is the movement of the diaphragm and the heartbeat, together with the arterial pulses of the bloodstream that contribute to an appropriate flow of lymph [12-13]. In our department, we do not perform AMLPM on patients with COPD. Instead, we recommend approaches which are gentle and of osteopathic derivation, osteopathic manipulative treatment (OMT), which have already been clinically tested for pain reduction, are without side effects, and achieve precocious clinical stability [48-49]. The goal is to improve the flow of the lymph and thus provide space to the tissues; our aim is not to increase the number of lymphocytes. By improving space for movement, the behavior of liquids changes, thus creating improved mechanotransductive interactions [12]. Our approach is standardized and with the patient seated; the operator remains behind the patient. The first approach is for the thoracic outlet: “The operator stands behind the patient, resting his/her hands on the thoracic outlet, with the index and middle fingers on the clavicle and the thumbs up to cervical-7 (C7), facing each other, while the ring finger and little finger are placed on the anterior area of the chest” [48]. We approach the thoracic strait because it is an important area in which the venous and lymphatic systems connect. The second phase concerns the mediastinal area: “In the second phase, the operator places one hand on the anterior area of the sternum, while the other hand is parallel on the patient’s back” [48]. By reducing the mediastinal musculoskeletal tension, one should positively affect the movement of the viscera inside the ribcage. The third and last phase is for the diaphragm muscle: “involve the operator’s hands being placed on the anterolateral area on the costal diaphragm” [48]. By giving the diaphragm the possibility of encountering less costal resistance, our technique should improve its excursion capacity and favor the natural respiratory lymphatic pump. A previous study has shown manually working on the muscular structure of the diaphragm can improve the expression of movement [45]. The operator does not induce movement, but rather follows the intrinsic movements that can be detected palpatorially. The technique ends when the movements perceived by the operator are expressed in a harmonious way. Generally, the duration of the procedure is only 15 minutes [48-49]. We do not yet know if this procedure affects deep lymphatic drainage, but by reducing musculoskeletal tension, it is increasingly possible that the patient can breathe with greater ease. With these premises, I can strongly hypothesize a better lymphatic flow in both the superficial and deep layers. Further studies are now needed to verify if this OMT protocol is able to positively influence the flow of the lymphatic system of the mediastinum.

## Conclusions

The article reviewed the anatomy of the lymphatic system, the lymphatic organization of the mediastinum, and discussed what happens to the lymphatic vessels and nodes in the presence of COPD. I have included and elaborated upon, previous literature relating to lymphatic pumping, in animals and patients. The ultimate goal of the article is to be able to offer a new manual approach to the patient, thanks to the clinical analysis of lymphatic anatomy in terms of physiology and pathology, and through a critical review of past research on the same topic. My proposed hypothesis is based on an already registered osteopathic approach to reduce mediastinal pain. Further studies will now be needed to verify if my OMT protocol is able to positively influence the flow of the lymphatic system in the mediastinum.
